# Endoscopic submucosal dissection is a safe and effective method for the treatment of duodenal papillary neuroendocrine tumor involving the pancreatic duct orifice

**DOI:** 10.1055/a-2786-0619

**Published:** 2026-03-09

**Authors:** Yi Yang, Haibin Zhang, Kang Fang, Li Zhang, Meidong Xu

**Affiliations:** 1 66324 Endoscopy Center, Department of Gastroenterology, Shanghai East Hospital, Shanghai, China; 2 Department of Pathology, Shanghai East Hospital, Shanghai, China

**Fig. 1:**
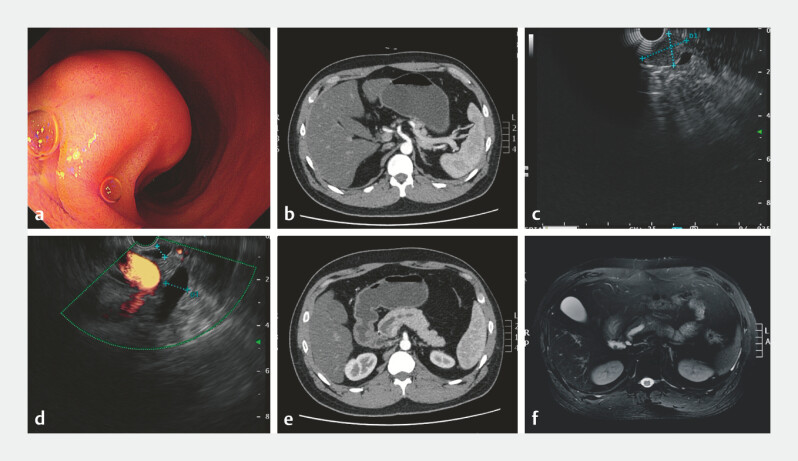
Preoperative relevant imaging examinations. **a** Endoscopic morphology of tumor of duodenal papilla. **b** Computed tomography (CT) showing the nodular soft tissue of 19 mm in diameter located in the duodenal papilla. **c** Endoscopic ultrasound (EUS) showing a hypoechoic mass of 20 mm in diameter. **d** EUS showing the dilated main pancreatic duct (an internal diameter of about 1cm). **e** A CT scan showing the dilated main pancreatic duct. **f** A magnetic resonance imaging (MRI) scan showing the dilated main pancreatic duct.

We report the case of a 32-year-old male patient with the duodenal papillary neuroendocrine tumor (NET) that was found during screening gastroscopy and computed tomography (CT; Fig. 1 **a, b** ). Endoscopic ultrasound (EUS) showed a hypoechoic mass of 20 mm in diameter ( Fig. 1 **c** ). The lesion involved the pancreatic duct orifice, with main pancreatic duct dilation (an internal diameter of about 10 mm; Fig. 1 **d** ). Same CT and magnetic resonance imaging revealed pancreatic duct dilation ( Fig. 1 **e, f** ).

**Fig. 2:**
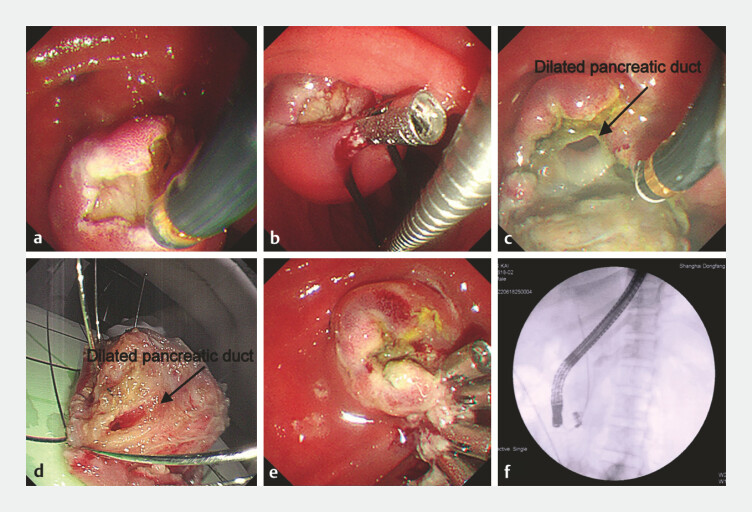
The process of the endoscopic submucosal dissection (ESD) of the duodenal papilla NET. **a** Incision along the margin of the tumor using the golden knife. **b** Traction assistance using the clips and snare. **c** Transection of the main pancreatic duct with pancreatic duct dilation. **d** The resected tumor with the dilated main pancreatic duct. **e** Closing the wound surface using metallic clips. **f** Placement of one stent in the common bile duct (7 F*10 cm straight plastic stents) and one in the main pancreatic duct (5 F*9 cm single pigtail plastic stent). NET, neuroendocrine tumor.

**Fig. 3:**
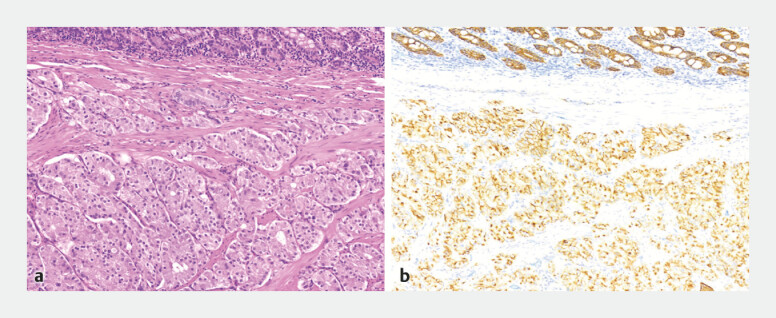
Postoperative pathology. **a** Hematoxylin–eosin (H&E)-stained and **b** immunohistochemical staining for CgA views of the resected specimen.

We decided to perform endoscopic submucosal dissection (ESD) of the lesion ( Video 1 ). After submucosal injection of glycerin fructose and indigo carmine showed adequate lifting, we made a marginal incision and gradually dissected along the base with the help of the metal clip and snare ( Fig. 2 **a, b** ). After transection of the pancreatic duct, intraoperative findings revealed tumor involving the pancreatic duct orifice with pancreatic duct dilation ( Fig. 2 **c, d** ). Electrocoagulation was used for hemostasis and the metal clips were used to close the wound surface ( Fig. 2 **e** ). Endoscopic retrograde cholangiopancreatography was performed to place the stents in the common bile and the pancreatic duct ( Fig. 2 **f** ). There were no complications during the perioperative period. The postoperative pathology confirmed the NET G1 stage ( Fig. 3 **a, b** ). At a 3-month follow up, gastroscopy revealed a regular scar, without macroscopic evidence of tissue residues or signs of recurrence.

Endoscopic dissection of duodenal papillary neuroendocrine tumors involving the pancreatic duct.Video 1

Duodenal papillary NETs are exceedingly rare, accounting for less than 2% of all ampullary tumors 1 , particularly those involving the pancreatic duct orifice. There are currently no guidelines for managing ampullary NETs 2 . Surgery, such as pancreaticoduodenectomy, is the traditional treatment for such cases 3 . We reported for the first time that ESD was performed for the duodenal papillary NET involving the main pancreatic duct orifice, which was a safe and feasible treatment with less invasive. In the future, larger clinical studies and long-term follow-up are needed to validate its safety and efficacy.

Endoscopy_UCTN_Code_TTT_1AO_2AL
